# Inactivated Vaccines Against SARS-CoV-2: Neutralizing Antibody Titers in Vaccine Recipients

**DOI:** 10.3389/fmicb.2022.816778

**Published:** 2022-03-10

**Authors:** Haiying Zhang, Yuyuan Jia, Ying Ji, Xu Cong, Yan Liu, Ruifeng Yang, Xiangsha Kong, Yijun Shi, Ling Zhu, Zhenyu Wang, Wei Wang, Ran Fei, Feng Liu, Fengmin Lu, Hongsong Chen, Huiying Rao

**Affiliations:** Peking University People’s Hospital, Peking University Hepatology Institute, National Clinical Research Center for Infectious Disease, Beijing Key Laboratory of Hepatitis C and Immunotherapy for Liver Diseases, Beijing International Cooperation Base for Science and Technology on NAFLD Diagnosis, Beijing, China

**Keywords:** inactivated SARS-CoV-2 vaccine, SARS-CoV-2 NAbs, SARS-CoV-2 IgM antibody, SARS-CoV-2 IgG antibody, serological test

## Abstract

**Background:**

Although effective vaccines have been developed against coronavirus disease 2019 (COVID-19), the level of neutralizing antibodies (NAbs) induced after vaccination in the real world is still unknown. The aim of this work was to evaluate the level and persistence of NAbs induced by two inactivated COVID-19 vaccines in China.

**Methods:**

Serum samples were collected from 1,335 people aged 18 years and over who were vaccinated with an inactivated COVID-19 vaccine at Peking University People’s Hospital from January 19 to June 23, 2021, for the detection of anti-severe acute respiratory syndrome coronavirus 2 (SARS-CoV-2) antibodies.

**Results:**

The positive rate for NAbs against SARS-CoV-2 was 79–91% from the first month to the second month after the second vaccine dose. The gradual decline in positivity rate for NAb response was observed from 78% at 3 months post-vaccination to 0% at 12 months post-vaccination. When there was a 21-day interval between the two doses of vaccine, the NAb positivity rate was 0% 6 months after the second dose. NAb levels were significantly higher when the interval between two doses were 3–8 weeks than when it was 0–3 weeks (χ2 = 14.04, *p* < 0.001). There was a linear correlation between NAbs and IgG antibodies in 1,335 vaccinated patients. NAb levels decreased in 31 patients (81.6%) and increased in 7 patients (18.4%) over time in the series of 38 patients after the second vaccination. The NAb positivity rate was significantly higher in 18- to 40-year-old subjects than in 41- to 60-year-old subjects (*t* = −*1*.*959*, *p* < *0*.*01*; *t* = *0*.*839*, *p* < *0*.*01*).

**Conclusion:**

The NAb positivity rate was the highest at the first and second month after the second dose of vaccine, and gradually decreased over time. With a 21-day interval between two doses of vaccine, neutralizing antibody levels persisted for only 6 months after the second dose of vaccine. Therefore, a third vaccine dose is recommended. Our results suggest that in cases in which NAbs cannot be detected, IgM/IgG antibodies can be detected instead. The level of NAbs produced after vaccination was affected by age but not by sex. Our results suggest that an interval of 21 to 56 days between shots is suitable for vaccination.

## Introduction

The novel severe acute respiratory syndrome coronavirus 2 (SARS-CoV-2) is the causative agent of novel coronavirus pneumonia [coronavirus disease 2019 (COVID-19)], which has caused a global pandemic. A safe, effective, and rapidly deployable vaccine is an effective global measure to control the spread of an outbreak. As virus mutations have been reported recently, an effective vaccine against SARS-CoV-2 is urgently needed to control the global COVID-19 pandemic. Currently, more than 280 vaccine candidates are in development worldwide, of which 23 are in phase three clinical trials (World Health Organization, 2021). Inactivated vaccines, such as the influenza vaccine, have been widely studied and used to prevent respiratory tract infection due to their good safety (Kyriakidis et al., 2021). At present, inactivated vaccines that have been approved for marketing in China have been proven to be effective and safe through clinical trials (Xia et al., 2020; Al Kaabi et al., 2021; Baden et al., 2021). However, after the marketing of vaccines, the clinical protective effect in the case of large-scale vaccination should be observed, and the protective persistence of vaccines should be studied. A phase 3 clinical trial of inactivated vaccines in China evaluated the efficacy and persistence of immunization by detecting neutralizing antibody (NAb) titers (Al Kaabi et al., 2021). Currently, China has launched inactivated SARS-CoV-2 vaccines from the Beijing Institute of Biological Products (Sinopharm), Wuhan Institute of Biological Products (Sinopharm), and Sinovac. According to the official website of the National Health Commission, as of November 15, 2021, a total of 2,396,045,000 doses of novel coronavirus vaccine have been administered as reported in 31 provinces (autonomous regions and municipalities under the central government) and by Xinjiang Production and Construction Corps (Bureau for Disease Control and Prevention, 2021).

However, the level of production of anti-SARS-CoV-2 NAbs induced by inactivated vaccines has not been confirmed and needs to be evaluated in large samples. Serological testing for NAbs against SARS-CoV-2 is important for assessing vaccine and treatment responses and comparing multiple drug candidates. In addition, reliable serological testing is needed to understand the true impact of COVID-19 through sero-epidemiological studies, as most cases are asymptomatic, and mild cases go largely undetected (Long et al., 2020). Virus neutralization tests (NTs) are the gold standard for the detection of NAbs, but they are complex and require BSL3 facilities. Previously published articles reported that serological tests, such as the pseudovirus neutralization test, can replace conventional virus neutralization test with BSL-3 requirement, as well as to determine vaccine efficacy during clinical trials and after mass vaccination (Tan et al., 2020; Müller et al., 2021).

In contrast, alternative fully automated chemiluminescence instruments offer the possibility of high-throughput testing in the laboratory. This study will only evaluate the NAb levels in the general population after two doses of vaccination. Correlation studies will be carried out to find out the relationships of NAb levels with age and gender of a patient, if any. This study provides an important reference basis for vaccine research and development, drug treatment, epidemiology, and immune surveillance.

## Materials and Methods

### Study Population

Serum samples were collected from 1,335 patients aged 18 years and over who received one dose of inactivated vaccine or two doses of inactivated vaccine at Peking University People’s Hospital from January 19 to June 23, 2021. They were divided into two groups: 244 patients received one dose of an inactivated vaccine, and 1,091 patients received two doses of an inactivated vaccine. All patients had no history of COVID-19 infection. In this study, the participants received two types of inactivated vaccines. The two vaccines are made by different companies. One vaccine was designed by the Beijing Institute of Biological Products Co., Ltd., and the Wuhan Institute of Biological Products Co., Ltd. They both belong to the China National Biotec Group Company Limited. Two SARS-CoV-2 strains (WIV04 and HB02) were isolated from two patients in Jinyintan Hospital, Wuhan, China, and separately used to develop the two vaccines (referred to as WIV04 and HB02 vaccines hereafter). The other vaccine was manufactured by Sinovac Life Sciences (Beijing, China). CoronaVac is an inactivated vaccine candidate against COVID-19, created from African green monkey kidney cells (Vero cells) that have been inoculated with SARS-CoV-2 (CN02 strain). Among the 1,335 cases, 1,025 received the Sinovac vaccine and 310 received the WIV04 and HB02 vaccine (Al Kaabi et al., 2021; Zhang et al., 2021). This study was approved by the ethics committee of Peking University People’s Hospital. Participants provided written informed consent to take part in the study.

### Measurement of Anti-severe Acute Respiratory Syndrome Coronavirus 2 Antibody Levels

The indirect method was used for antibody detection. S- IgG and S- IgM levels were detected using a chemiluminescence assay kit. Magnetic particles were coated with 2019-nCoV antigen, and anti-human IgG antibody was labeled with horseradish peroxidase to prepare enzyme conjugals. The complex catalyzed the conversion of the luminescence substrate to give off photons, and the luminescence intensity was proportional to the content of anti-SARS-CoV-2 IgG antibody. The serum sample size required was 80 μl. When the signal-to-cut-off (S/CO) ratio was ≥ 1.00, the result was positive; when the S/CO ratio was < 1.00, the result was negative. An Autolumo A2000 PLUS automatic chemiluminescence immunoanalyzer from Zhengzhou Antu Biological Engineering Co., Ltd. (Autobio Diagonostic) was used as the supporting instrument for the above reagents.

### Measurement of Anti-severe Acute Respiratory Syndrome Coronavirus 2-Neutralizing Antibody Levels

The neutralization antibodies against RBD proteins of SARS-CoV-2 in serum specimens were detected by a chemiluminescence method according to the instructions of the manufacturer (Autobio Diagonostic), respectively. Antibody levels ≥ 30 AU/ml are reactive (positive), and the results < 30 AU/ml are negative. The specific interaction of ACE2 and RBD protein can be neutralized by SARS-CoV-2-neutralizing antibodies. SARS-CoV-2-specific neutralizing antibodies in the sample bind to the HRP-labeled RBD antigen, which neutralizes the interaction of ACE2 coated on the microparticles and the RBD antigen. The HRP-labeled RBD antigen not neutralized by the SARS-CoV-2-specific neutralizing antibodies forms a complex with ACE2 on the microparticles. Chemiluminescent substrate is added, and the complex catalyzes the substrate, resulting in a chemiluminescent reaction. The serum sample size required was 80 μl. When the concentration value of the sample was ≥ 30.00 AU/ml, the result was judged as positive, and when the concentration value was < 30.00 AU/ml, the result was judged as negative. An Autolumo A2000 PLUS automatic chemiluminescence immunoanalyzer from Zhengzhou Antu Biological Engineering Co., Ltd. (Autobio Diagonostic) was used as the supporting instrument for the above reagents.

### World Health Organization Standard for Detection of Anti-severe Acute Respiratory Syndrome Coronavirus 2-Neutralizing Antibodies

Using the World Health Organization (WHO) first-generation international standard (IS) for antibodies against SARS-CoV-2 (NIBSC Code: 20/136), the international unit concentration (IU/ml) of NAbs was determined, the reference was lyophilized powder, with 0.25 ml of standard substance per tube, and the original concentration was 1,000 IU/ml. The theoretical dilution concentrations were 500, 250, 125, 72.5, 36.25, and 18.125 IU/ml. Each sample was tested five times in parallel, and the mean was calculated.

### Quality Control

Each test batch of the above two kits included negative quality control products and positive quality control products for SARS-CoV-2 antibodies/NAbs.

### Statistical Analysis

SPSS 21.0 was used for statistical analysis. The age of patients was measured and was found to be normally distributed, denoted by X ± S. The χ2 test was used for statistical analysis of the count data between the two groups. Statistical analysis of measurement data between the two groups was performed by a *t*-test, and *p* < 0.050 was considered statistically significant.

## Results

### Analysis of the Clinical Data of 1,335 Patients

The clinical characteristics of the 1,335 patients were described. Among the 1,335 vaccinated people, there were 644 men and 691 women aged 36.27 ± 12.39 years, and the sex ratio (male/female) was 0.93:1. Among them, there were 243 cases in the one-dose vaccine group (age 35.26 ± 11.87 years old), including 120 men and 123 women, with a sex ratio (male/female) of 0.98:1. There were 1,092 patients (36.50 ± 12.49 years old) in the two-dose vaccine group, including 524 men and 568 women, with a sex ratio of 0.92:1. For the 570 patients, the interval between the two doses was 21 days, and for the remaining 522, the interval between the two doses was 0 to 77 days, with a median interval of 24 days. To assess the change in NAbs over time in people who completed the two-dose vaccine, we calculated the median number of days between completing the two-dose vaccine and testing for SARS-CoV-2 antibodies, which was 50 days (0–332 days). The median number of days between the anti-SARS-CoV-2 antibody test date and the second vaccine dose was 50 days (0–332 days) (Table 1).

**TABLE 1:** Clinical characteristics of adults vaccinated with the two inactivated vaccines.

	No. (%)			
	
	Group with a 21-day interval between the two doses(*n* = 570)	Received two vaccine doses (*n* = 1,092)	Received one vaccine dose (*n* = 243)	Vaccine group (*n* = 1,335)
Age, (mean ± SD)	36.11 ± 11.95	36.50 ± 12.49	35.26 ± 11.87	36.27 ± 12.39
Age				
> 60 years	16 (2.8)	46 (4.2)	12 (4.9)	58 (4.3)
18–60 years	554 (97.2)	1,046 (95.8)	231 (95.1)	1,277 (95.7)
Sex				
Male	270 (47.4)	524 (48.0)	120 (49.4)	644 (48.2)
Female	300 (52.6)	568 (52.0)	123 (50.6)	691 (51.8)
NAb positive	436 (76.5)	844 (77.3)	44 (18.1)	888 (66.5)
Anti-SARS-CoV-2 IgG antibody positive	482 (84.6)	911 (83.4)	76 (31.3)	987 (73.9)
Anti-SARS-CoV-2 IgM or IgG antibody positive	534 (93.7)	1,019 (93.3)	84 (34.6)	1,103 (82.6)
Interval between two vaccine doses (median days)	21	21 (0–77 days)	/	/
Interval between antibody test date and second vaccine dose (median days)	54 (0–332 days)	50 (0–332 days)	/	/
21 days between two vaccine doses	570 (100)	570 (52.20)	/	570 (42.70)

*SD, standard deviation; NAb, neutralizing antibody; SARS-CoV-2, severe acute respiratory syndrome coronavirus 2.*

### World Health Organization Standard Detection of Anti-severe Acute Respiratory Syndrome Coronavirus 2 Neutralizing Antibodies

The performance of anti-SARS-CoV-2 NAbs reagent was verified with WHO standard product. WHO SARS-COV-2 NAbs standard was detected with NAbs reagent. The original concentration of WHO SARS-COV-2 NAbs standard was 1,000 IU/ml. The mean value of our detection results was 1,035 AU/ml, and the coefficient of variation was 1.3%. The coefficient of variation between the test results and the true values of NAbs of the WHO standard at theoretical concentrations of 500, 250, 125, 72.5, 36.25, and 18.125 IU/ml were all lower than the WHO IS of 3%.

### Changes in Neutralizing Antibody Positivity Rate/Levels Each Month After the Second Dose of Inactivated Vaccine

**FIGURE 1:**
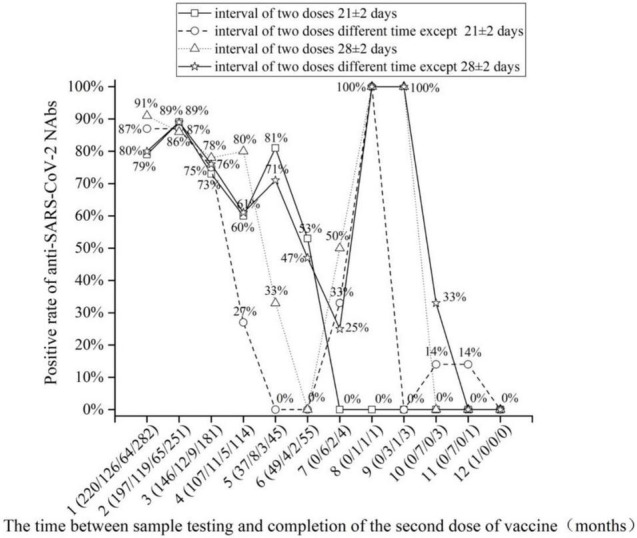
The positive rate of anti-severe acute respiratory syndrome coronavirus 2 (SARS-CoV-2) neutralizing antibody (NAb) detection with the increase in months after the completion of two doses of vaccine.

**FIGURE 2:**
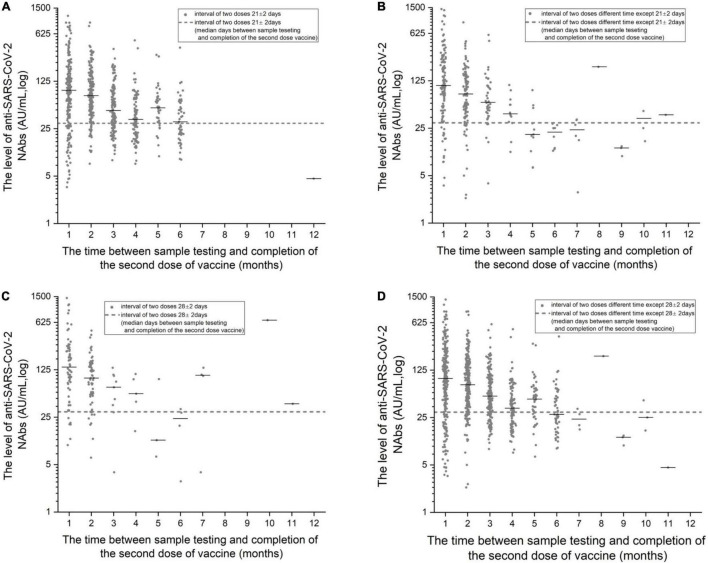
**(A)** The detection level of anti-SARS-CoV-2 NAb with the increase in months after the completion of the two doses of vaccine at 21 ± 2-day intervals. **(B)** The detection level of anti-SARS-CoV-2 NAb with the increase in months after the completion of the two doses of vaccine at intervals other than 21 ± 2-days. **(C)** The detection level of anti-SARS-CoV-2 NAb with the increase in months after the completion of the two doses of vaccine at 28 ± 2-day intervals. **(D)** The detection level of anti-SARS-CoV-2 NAb with the increase in months after the completion of the two doses of vaccine at intervals other than 28 ± 2-days.

The monthly changes in NAb levels and positivity rates were evaluated after the completion of two doses of vaccine. In Figures 1, 2, the 1,092 patients who received two doses of vaccine were divided into two doses 21 ± 2 days apart and the non-21± 2 days group according to the 21 ± 2 day interval between the two doses of vaccine; the 1,092 patients who received two doses of vaccine were divided into two doses 28 ± 2 days apart and the non-28 ± 2 days group according to the 28 ± 2-day interval between the two doses of vaccine. Figures 1, 2 depict the changes in NAb positivity rate/level each month after two doses of vaccination. Among the 1,092 patients in the two doses of the inactivated vaccine group, we observed that from the second dose after vaccination from 1 month to the 2nd month, the anti-SARS-CoV-2 NAb positivity rate was 79–91%; from the 3rd month to the 12th month, the NAb positivity rate dropped to less than 33%. In Figure 2, from the 1st month to the 2nd month after the second dose, the level of anti-SARS-CoV-2 NAb was higher than the 3rd month to the 12th month. It is, thus, recommended to test for NAb levels from the 1st month to the 2nd month after the second dose is completed, when the NAb level is the highest (Figures 1, 2). In Figure 2A, when the interval between two doses was 21 ± 2 days, the NAb positivity rate at the 6th month (∼53%) after the last dose was significantly lower than that at the 5th month (∼81%), with statistical significance (χ2 = 7.286, *p* < 0.05). Subjects were NAb negative 7 to 12 months after vaccination, and NAb positivity lasted only for 6 months (Table 2). In Figure 2C, when the interval between two doses was 28 ± 2 days, the NAb positivity rate in the 5th month (∼33%) after the last dose was significantly lower than that in the 4th month (∼80%), with no statistical significance (χ2 = 1.742, *p* > 0.05) (Table 3). NAb was negative from the 7th to 9th month, but the positivity rate of NAb increased to 100% in the 10th and 11th month. However, there was only one person per month in the 10th and 11th month, and this one person was NAb positive, so the number was too small to be representative (Figure 2C). In Figure 2B, the non-21-day interval between vaccine and NAb positivity rate was 100% in 8th and 11th months after the second vaccination, but in both months, there was only one person per month, so the numbers were small. In Figure 2D, the non-28-day vaccination interval, the NAb positivity rate was 100% 8 months after the second vaccination, but in this month, there was only one person, and the number was small. So it is not representative.

**TABLE 2:** Percentage of vaccinee positive for NAb at different months at 21 ± 2-day intervals.

	NAb positive n (%)	NAb negative n (%)	Total	*p*-Value
Fifth month	30 (81.08)	7 (18.92)	37	< 0.05
Sixth month	26 (53.06)	23 (46.94)	49	
Total	56	30	86	

**TABLE 3:** Percentage of vaccinee positive for NAb at different months at 28 ± 2-day intervals.

	NAb positive *n* (%)	NAb negative *n* (%)	Total	*p*-Value
Fourth month	4 (80.00)	1 (20.00)	5	> 0.05
Fifth month	1 (33.33)	2 (66.67)	3	
Total	5	3	8	

### Anti-severe Acute Respiratory Syndrome Coronavirus 2 Antibody Levels Generated at Different Time Intervals Between Two Doses of Vaccine

We studied the difference in the NAb positivity rate of the two vaccine populations at different time intervals. The data in Table 2 refer to a total of 1,088 people who completed two doses of vaccine with an interval of 0–3 and 3–8 weeks. A total of 1,092 people received two doses of vaccine, except for four who received two doses more than 60, 64, 73, 77 days apart, and 1,088 who received two doses within a range of 0 to 8 weeks. The novel Coronavirus Vaccine Technical Guide (first edition) published on the website of the Central People’s Government of the People’s Republic of China states that the recommended interval between two doses of the novel coronavirus inactivated vaccine is not less than 3 weeks, and the second dose should be completed as soon as possible within 8 weeks. Therefore, we divided it into 0–3 and 3–8 weeks. The interval between the two doses of vaccine was from 0 to 77 days, and the positivity rates of NAb generation at 3- to 8-week intervals between the two doses of vaccine were very high (80.8%). The positivity rates of anti-SARS-CoV-2 NAbs were significantly different between the two groups (0–3 and 3–8 weeks) (χ2 = 14.04, *p* < 0.001) (Table 4).

**TABLE 4:** Correlation between NAbs and the time interval between two coronavirus disease 2019 (COVID-19) vaccine doses.

Interval between two vaccines	NAb positive *n* (%)	NAb negative *n* (%)	Total	*p*-Value
0–3 weeks	68 (63.0)	40 (37.0)	108	< 0.01
3–8 weeks	773 (80.8)	207 (19.2)	980	
Total	841	247	1,088	

### Changes in Neutralizing Antibody Levels After Two Severe Acute Respiratory Syndrome Coronavirus 2 Vaccine Doses in the Same Person

**FIGURE 3:**
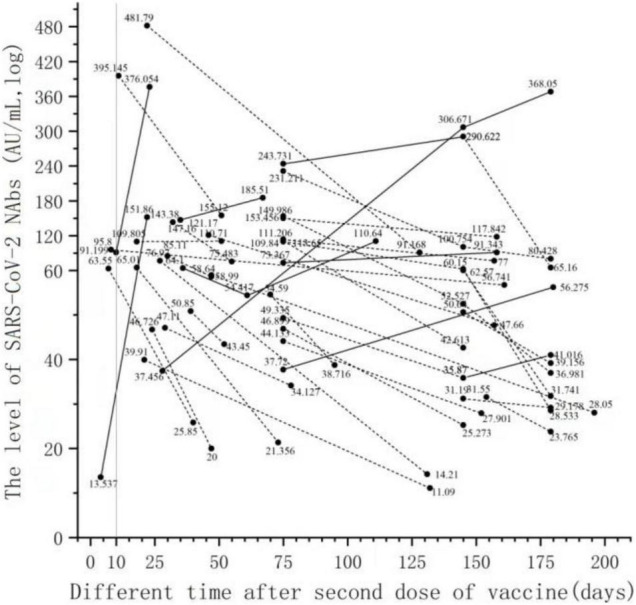
Changes in NAb levels over time in serial serum samples after the second dose of vaccine. Only 38 people had a series of sera after completing two doses of the vaccine. Thirty-eight people have two time points for detecting NAbs after completing two doses of vaccine injection, and the line connecting the points belongs to the sample of the same subject.

The serum series after the two doses of the vaccine were used to assess the changes in NAb levels over time. Only 38 people had a series of sera after completing two doses of the vaccine. In Figure 3, 38 people have two time points for detecting NAbs after completing two doses of vaccine injection, and the line connecting the points belongs to the sample of the same subject. NAb levels decreased in 31 patients (81.6%) and increased in 7 patients (18.4%) over time in the series of 38 patients after the second vaccination (Figure 3). There were six men and one woman in the group with elevated NAb levels (7 patients). There were 13 men and 18 women in the group with decreased NAb levels (31 patients). The proportion of men in the group with increased NAb levels was significantly higher than that in the group with decreased NAb levels (χ2 = 4.378, *p* < 0.05). There was no significant difference in age between the group with increased NAb levels and the group with decreased NAb levels (*t* = −0.034, *p* > 0.05).

### Association Between the Presence of Anti-severe Acute Respiratory Syndrome Coronavirus 2 Antibodies and Neutralizing Antibody After Vaccination

**FIGURE 4:**
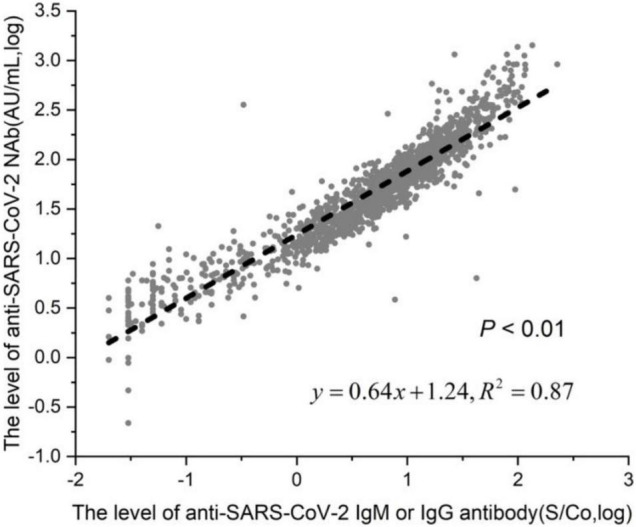
Linear correlation of anti-SARS-CoV-2 NAb and antiSARS-CoV-2 antibody. Among the 1,335 patients, 1,103 were in the anti-SARS-CoV-2 IgM or IgG positive group, and 232 were in the negative group.

We are studying the association between the presence of anti-SARS-CoV-2 antibodies and of NAb in 1,335 vaccinated individuals. Among the 1,335 patients, 1,103 were anti-SARS-CoV-2 IgM or IgG positive, and the positivity rate was 82.6% (1,103/1,335); 888 were anti-SARS-CoV-2 NAb positive, and the positivity rate was 66.5% (888/1,335). Of the 888 NAb-positive patients, 886 were anti-SARS-CoV-2 IgM or IgG positive, and the positivity rate is 66.4% (886/1335). Among the 1,335 patients, the positivity rate of SARS-CoV-2 Nab in the SARS-CoV-2 antibody-positive group (80.1%) was significantly higher than the SARS-CoV-2 NAb positivity rate in the SARS-CoV-2 antibody-negative group (19.9%), and the difference was statistically significant (Table 5). There was a linear correlation between anti-SARS-CoV-2 NAb and anti-SARS-CoV-2 IgM or IgG titer among the 1,335 patients (Figure 4).

**TABLE 5:** Contingency tables between anti-SARS-CoV-2 NAbs and anti-SARS-CoV-2 antibodies.

	NAb positive *n* (%)	NAb negative *n* (%)	Total	*p*-Value
Anti-SARS-CoV-2 IgM or IgG positive	886 (80.1)	219 (19.9)	1,103	< 0.001
Anti-SARS-CoV-2 IgM or IgG negative	2 (1.7)	228 (98.3)	232	
Total	888	447	1,335	

### Differences in the Levels of Neutralizing Antibodies Against Severe Acute Respiratory Syndrome Coronavirus 2 Between Groups Receiving One Dose of Vaccine and Two Doses of Vaccine

The difference in the positivity rate of NAbs between the one-dose (243/1,335 subjects) and two-dose vaccine groups (1,092/1,335 subjects) was evaluated. Each group was further stratified by the time at which antibodies were measured after vaccination, specifically less than 2 months or more than 2 months after the last dose of the vaccine. Among the 1,335 patients, 1,092 patients received two doses of vaccine, and 243 patients received one dose of vaccine. Among the 1,092 patients in the two-dose vaccine group, the time of NAb detection was less than 2 months after the completion of the last dose of vaccine in 562 patients, and the positivity rate of SARS-CoV-2 antibody was 84.89% (Table 6). The NAb detection time of 282 patients was more than 2 months after finishing the last dose of vaccine, and the positivity rate of SARS-CoV-2 antibody was 65.58% (Table 7). Among the 243 patients in the single-dose vaccine group, 43 patients tested NAb less than 2 months after finishing the last dose of vaccine, and the positivity rate of SARS-CoV-2 antibody was 18.14% (Table 6). The NAb detection time of one patient was more than 2 months after finishing the last dose of the vaccine, and the positivity rate of SARS-CoV-2 antibody was 16.67% (Table 7). In the group less than 2 months after the last dose of vaccine, the positivity rate of SARS-CoV-2 antibody in the two-dose vaccine group (84.89%) was significantly higher than that in the single-dose vaccine group (18.14%), and the difference was statistically significant (χ2 = 353.327, *p* < 0.001) (Table 6). In the group more than 2 months after the last dose of vaccine, the positivity rate of SARS-CoV-2 antibody in the two-dose vaccine group (65.58%) was significantly higher than that in the single-dose vaccine group (16.67%) (χ2 = 6.216, *p* < 0.05) (Table 7).

**TABLE 6:** Contingency tables of doses and NAbs (The time between the last dose of vaccine and testing was less than 2 months).

	NAb positive *n* (%)	NAb negative *n* (%)	Total	*p*-Value
Received one vaccine dose	43 (18.14)	194 (81.86)	237	< 0.001
Received two vaccine doses	562 (84.89)	100 (15.11)	662	
Total	605	294	899	

**TABLE 7:** Contingency tables of doses and NAbs (The time between testing and the last dose of vaccine should be at least 2 months).

	NAb positive *n* (%)	NAb negative *n* (%)	Total	*p*-Value
Received one vaccine dose	1 (16.67)	5 (83.33)	6	< 0.05
Received two vaccine doses	282 (65.58)	148 (34.42)	430	
Total	283	153	436	

### Contingency Tables Between Age and Sex and the Level of Neutralizing Antibodies Against Severe Acute Respiratory Syndrome Coronavirus 2 in the Two-Dose Vaccine Group

**FIGURE 5:**
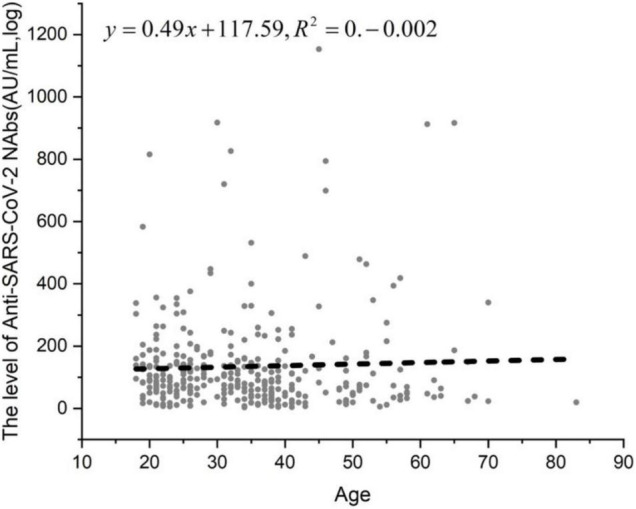
The linear correlation between age and anti-SARS-CoV-2 NAb level. In the two-dose vaccine group and the 21-day interval between the two vaccines, the difference in NAbs in different age or gender groups was observed. A total of 329 people who were tested 1 to 2 months after receiving the second dose were tested and divided into three groups.

In the two-dose vaccine group and the 21-day interval between the two vaccines, the difference in NAbs in different age or gender groups was observed. A total of 329 people who were tested 1 to 2 months after receiving the second dose were tested and divided into three groups based on age: 18–40 years old, 41–60 years old and > 60 years old. The positivity rates of NAbs in the three groups were 85.60, 77.78, and 78.57%, respectively (Table 8). The NAb positivity rate was higher in 18- to 40-year-old subjects than in 41- to 60-year-old subjects, but there was no significant difference (χ2 = 2.502, *p* > 0.05). The NAb positivity rate was higher in 18- to 40-year-old subjects than in > 60-year-old subjects, but there was no significant difference (χ2 = 0.045, *p* > 0.05). The NAb positivity rate was higher in 41- to 60-year-old subjects than in > 60-year-old subjects, but there was no significant difference (χ2 = 0.004, *p* > 0.05). The NAb titer in the 18- to 40-year-old group was significantly lower than that in the 41- to 60-year-old group (*t* = −0.839, *p* < 0.01). The NAb titer was slightly lower in the 18- to 40-year-old group than in the > 60-year-old group, but there was no significant difference (*t* = −1.959, *p* < 0.01). The NAb titer produced in the 41- to 60-year-old group was lower than that in the > 60-year-old group, with no significant difference (*t* = −0.960, *p* > 0.05). There was a poor linear correlation between age and antibody titer among the 329 patients (Figure 5).

**TABLE 8:** Percentage of vaccinee positive for NAb by age.

	NAb positive *n* (%)	NAb negative *n* (%)	Total
18–40 years	208 (85.60)	35 (14.40)	243
41–60 years	56 (77.78)	16 (22.22)	72
> 60 years	11 (78.57)	3 (21.43)	14
Total	275	54	329

A total of 329 people who were tested 1 to 2 months after the second injection were divided into men and women groups. The NAb positivity rate in the men group (82.63%) was lower than that in the women group (84.57%), but there was no significant difference (χ2 = 0.224, *p* > 0.05). The NAb titer in the men group was lower than that in the women group, but there was no significant difference (*t* = −0.310, *p* > 0.05) (Table 9).

**TABLE 9:** Percentage of vaccinee positive for NAb by sex.

	NAb positive *n* (%)	NAb negative *n* (%)	Total	*p*-Value
Male	138 (82.63)	29 (17.37)	167	> 0.05
Female	137 (84.57)	25 (15.43)	162	
Total	275	54	329	

## Discussion

Our research shows that the NAb positivity rate was 66.5% among adults from the 1st to 12th months after receiving two doses of inactivated vaccine and from the 1st to 11th months after receiving one dose of vaccine. Among adults from the 1st to 12th month after receiving two doses of inactivated vaccine, the NAb positivity rate was 77.3%. In a phase three randomized clinical trial of adults vaccinated with Chinese vaccines, the protective efficacy of two whole-virus inactivated vaccines was 72.8 and 78.1% (Al Kaabi et al., 2021). In our study, two doses of an inactivated vaccine produced a NAb positivity rate in adults similar to that in previous studies, but one or two doses of an inactivated vaccine produced a NAb positivity rate of 66.5%, which is slightly lower than that in previous studies; the difference may be because of our study approach, as 18.1% of the population was vaccinated with only one dose, and previous research involved only two-inoculation vaccination. The phase 3 clinical trials reported in the literature have included mainly healthy and young people from the Middle East and other Asian countries, and the Middle East studies have been limited to the male population. Our study reflects the level of NAbs induced by vaccination in our population. Recently, interim results from phase 3 clinical trials of other vaccines, including two mRNA vaccines (BNT162b2 and mrN-1273) and three adenovirus-based vaccines [ChAdOx1 nCoV-19, GAM-COVID-19 (Sputnik V), and Ad26.COV2. S] have been published (Polack et al., 2020; Baden et al., 2021; Logunov et al., 2021; Sadoff et al., 2021; Voysey et al., 2021). The protective efficacy of the BNT162b2 (Pfizer-BioNTech) vaccine was reported to be 95.0%; the protective efficacy of the mrNA-1273 (Moderna) vaccine was 94.1%; that of ChAdOx1 nCoV-19 was 62.1 or 90.0%, depending on the dose regimen (Two standard doses or low doses followed by standard doses); that of the GAM-COVID-19 vaccine was 91.6%; and that of the AD26.COV2. S vaccine was 66.9%. These studies have mainly included people from Western countries; the proportion of elderly participants is higher, and our study cohort consists of the Chinese people, with ethnic and regional differences possibly leading to variable vaccine-induced NAb levels; therefore, our research can better reflect the actual situation of NAb production after vaccination in the Chinese people.

NIBSC in collaboration with WHO has developed the first-generation SARS-CoV-2 NAb for the standardization and harmonization of the protocols across the regions and laboratories. The IS is considered to be the highest level of biological material reference materials, for IS-based quantitative determination of the IU of biological activity, which can be compared with different laboratory analyses and make the results comparable to better define the analysis parameters (e.g., sensitivity of the test) and clinical parameters (such as the protection afforded by the antibody level). The anti-SARS-CoV-2 NAb IS will facilitate the standardization of serological assays for the detection of SARS-CoV-2 for use in vaccine studies to detect antibodies produced by human vaccination. Compared with the original WHO value, the coefficient of variation (CV) of our study was 1.3%, which met the WHO standard of less than 3%. Therefore, our NAb unit (AU/ml) is close to the IU/ml and will help to evaluate vaccine efficacy and data from epidemiological and immunological surveillance studies.

An important question regarding the efficacy and safety of COVID-19 vaccines is how long the protection they provide will last. Our study found that when the interval between doses was 21 ± 2 days, the NAb positivity rate was significantly lower at 6 months (53%) after the last dose than at 5 months (81%). Subjects were negative for NAb between 7 and 12 months after vaccination, and NAb lasted only for 6 months. Therefore, our study suggests that it is necessary to strengthen the immunization of the third dose of vaccine. A previous article found that healthy subjects showed sustained, but declining, NAb levels against SARS-COV-2 6 months after full vaccination with BNT162b2 vaccine (Terpos et al., 2021), but their vaccine, unlike ours, is not inactivated. Our results showed that the NAb positivity rate in the 1st to 2nd months after the second dose of vaccine was 79–91%, after which the positivity rate began to decline. The level of anti-SARS-CoV-2 NAbs drops from the 3rd month, and a third vaccine dose is recommended. Therefore, it is recommended to test the NAb level from 1 to 2 months after the second vaccine dose. The NAb positivity rate in the 5th month (33%) was significantly lower than that in the 4th month (80%) at the interval of 28 ± 2 days between two doses of vaccine. The positivity rate of NAb was 0% at 7–9 months, but increased to 100% in the 10th and 11th months. We analyzed the reasons and found that in the 10th and 11th months, there was only one person per month, and that person was NAb positive, so the number was too small to be representative. The non-21 ± 2-day interval between doses of vaccine and the NAb positivity rate was 100% in the 8th and 11th months after the second dose. The non-28 ± 2-day interval between doses of vaccine and the NAb positivity rate was 100% in 8 months after the second dose, but was not representative due to the small number of vaccinated people. The first 6 months after the second dose are typically large. The New England Journal of Medicine published persistent data on the persistence of the immune response stimulated by the candidate Moderna COVID-19 vaccine mRNA-1273 (Widge et al., 2021). The analysis showed that participants maintained high levels of antibodies binding to the novel coronavirus spike protein and of NAbs 90 days after the second vaccine dose. Even if the level of NAbs decreases slightly over time, mRNA-1273 still has the potential to provide lasting humoral immunity. In addition, a study published in Science (Dan et al., 2021) showed that even with low-plasma NAb activity triggered by novel coronavirus natural infection, the natural immunity could still trigger a robust memory B-cell response. When encountering the novel coronavirus again, these memory B cells can quickly produce targeted NAbs, which is crucial in maintaining long-term immunity to the novel coronavirus. Different studies on the levels of NAbs in recovered patients show that the persistence of NAbs in different patients is uneven. In some patients, the level of NAbs decreased significantly within a few months after recovery, so we studied the persistence of NAb levels triggered by COVID-19 vaccine.

Wang Huaqing, chief expert of the immunization program of the Chinese Center for Disease Control and Prevention, at a press conference under the Joint Prevention and Control Mechanism of the State Council, said that the interval between inactivated vaccine injections should be completed in less than 8 weeks^1^ (Peng, 2021).

In the two-dose vaccine group, the time interval was from 0 to 77 days, and the highest level of NAbs generated at the interval of 21–56 days between the two doses suggested that 21–56 days between the two doses was suitable for vaccination.

People aged 60 years and older have severe illness and high risk of death after contracting the novel coronavirus. Data from phase I/II clinical studies showed that the novel coronavirus vaccine was safe in this population (Walsh et al., 2020), and our study revealed that the average level of NAbs produced in the elderly population (>60 years old) was higher than that in the young (18–40 years old), which was different from previous studies (Walsh et al., 2020). Instead of receiving an inactivated vaccine in that study, the study population was vaccinated with an mRNA vaccine. Our study also showed that the NAb positivity rate in men in the two-dose group was substantially lower than in women, but the difference was not significant. Our study suggested that the level of NAbs produced after vaccination was affected by age, but not by sex.

The gold standard method for NAb quantitation is the NT (Balcells et al., 2021). Even if live or synthetic viruses are used to react with samples to detect the killing ability of NAbs against viruses in samples, published studies of the clinical efficacy evaluation of vaccines are now all using this method (Polack et al., 2020; Al Kaabi et al., 2021). However, this method has high requirements for testing and is not suitable for general medical testing institutions. Another kind of NAb detection is based on the immune reaction principle, through the specific antigen detection-corresponding NAbs, this method is suitable to promote use by general medical testing institutions. Because epidemic strains carry some point mutations at individual sites, there are mainly five or six mutations, and there are many NAbs targeting the S protein epitope. Individual point mutations will not lead to complete virus escape, which is why the vaccines are effective. Vaccines targeting the RBD area of the S protein induce the production of the main NAbs. Our NAb detection reagent is based on the S protein RBD, which facilitates detection in the same way that vaccines work. Our study showed a linear correlation between NAbs and anti-SARS-CoV-2 IgM/IgG antibodies in vaccinated individuals. Therefore, it is suggested that when NAbs cannot be detected, anti-SARS-CoV-2 IgM/IgG antibodies can be detected instead. A total of 89.6% (796/888) of these IgG-positive samples were also positive in NAbs in our study. This result differs from the results of a recently published study by Müller K et al., in which only 68.7% (158/230) of these IgG-positive samples were also positive in NTs (Müller et al., 2021). In that study, an ELISA was used to detect anti-SARS-CoV-2 antibodies, which is different from the chemiluminescence method used in our paper. Antibodies against SARS-CoV-2 produced by convalescent patients reduced the neutralization effectiveness against emerging mutations. Therefore, it is critical to monitor anti-SARS-CoV-2 antibody levels for vaccine surveillance and vaccine development (Tea et al., 2021).

There were some limitations of this study. First, the study did not include pregnant women or people under 18 years old. Therefore, the effectiveness of inactivated vaccines in these populations remains unknown. In future studies, the effective protective concentration of anti-SARS-CoV-2 NAbs should be determined through research; for example, the critical value of hepatitis B virus surface antibody is 10 mIU/ml.

In summary, the NAb positivity rate was the highest in the 1st and 2nd months after the second dose of vaccine, and gradually decreased over time. Therefore, a third vaccine dose is recommended. Our study shows that anti-SARS-CoV-2 IgM/IgG antibodies can be detected in cases in which NAbs cannot be detected. Twenty-one to 56 days (3–8 weeks) between the two doses was suitable for vaccination. Our study provides important insight into vaccination cycles, vaccination timing, and detection methods.

## Data Availability Statement

The original contributions presented in the study are included in the article/supplementary material, further inquiries can be directed to the corresponding author/s.

## Ethics Statement

The studies involving human participants were reviewed and approved by the Ethics Committee of the Peking University People’s Hospital. Written informed consent for participation was not required for this study in accordance with the national legislation and the institutional requirements.

## Author Contributions

HR and HC designed the experiments. HZ and YuJ performed the experiments. YiJ, XC, YL, and RY participated in the study. XK, YS, LZ, and ZW analyzed the data. WW, RF, FLi, and FLu have verified the underlying data. HR, HC, and HZ designed the experiments. All authors contributed to the article and approved the submitted version.

## Conflict of Interest

The authors declare that the research was conducted in the absence of any commercial or financial relationships that could be construed as a potential conflict of interest.

## Publisher’s Note

All claims expressed in this article are solely those of the authors and do not necessarily represent those of their affiliated organizations, or those of the publisher, the editors and the reviewers. Any product that may be evaluated in this article, or claim that may be made by its manufacturer, is not guaranteed or endorsed by the publisher.

## Footnotes

1
https://baijiahao.baidu.com/s?id=1700327464370422756&wfr=spider&for=pc
